# Wnt activity guides facial branchiomotor neuron migration, and involves the PCP pathway and JNK and ROCK kinases

**DOI:** 10.1186/1749-8104-4-7

**Published:** 2009-02-11

**Authors:** Valérie Vivancos, Ping Chen, Nathalie Spassky, Dong Qian, Alain Dabdoub, Matthew Kelley, Michèle Studer, Sarah Guthrie

**Affiliations:** 1 MRC Centre for Developmental Neurobiology, King's College, Guy's Campus, London, SE1 1UL, UK; 2 Department of Cell Biology, Emory University School of Medicine, 615 Michael St, Atlanta, GA30322, USA; 3 Biologie des Interactions Neurones/Glie, Unite Mixte de Recherche INSERM U-711 UPMC, Hôpital de la Saltpetriere, Batiment de Pharmacie, 75651 Paris, cedex 13, France; 4 Department of Surgery, Division of Otolaryngology, UCSD School of Medicine, Gilman Drive, La Jolla, CA 92093-0666, USA; 5 NIDCD/NIH, Porter Neuroscience Research Center, Convent Drive, Bethesda, MD 20892-3729, USA; 6 TIGEM, Via P Castellino 111, 80131 Naples, Italy

## Abstract

**Background:**

Wnt proteins play roles in many biological processes, including axon guidance and cell migration. In the mammalian hindbrain, facial branchiomotor (FBM) neurons undergo a striking rostral to caudal migration, yet little is known of the underlying molecular mechanisms. In this study, we investigated a possible role of Wnts and the planar cell polarity (PCP) pathway in this process.

**Results:**

Here we demonstrate a novel role for Wnt proteins in guiding FBM neurons during their rostral to caudal migration in the hindbrain. We found that *Wnt5a *is expressed in a caudal^high ^to rostral^low ^gradient in the hindbrain. Wnt-coated beads chemoattracted FBM neurons to ectopic positions in an explant migration assay. The rostrocaudal FBM migration was moderately perturbed in *Wnt5a *mutant embryos and severely disrupted in *Frizzled3 *mutant mouse embryos, and was aberrant following inhibition of Wnt function by secreted Frizzled-related proteins. We also show the involvement of the Wnt/PCP pathway in mammalian FBM neuron migration. Thus, mutations in two PCP genes, *Vangl2 *and *Scribble*, caused severe defects in FBM migration. Inhibition of JNK and ROCK kinases strongly and specifically reduced the FBM migration, as well as blocked the chemoattractant effects of ectopic Wnt proteins.

**Conclusion:**

These results provide *in vivo *evidence that Wnts chemoattract mammalian FBM neurons and that Wnt5a is a candidate to mediate this process. Molecules of the PCP pathway and the JNK and ROCK kinases also play a role in the FBM migration and are likely mediators of Wnt signalling.

## Introduction

Neuronal migration is a fundamental feature of the developing nervous system [1]. A striking migration is undertaken by the facial branchiomotor neurons (FBMs) of the mammalian embryonic hindbrain. In the mouse embryo, FBM neurons differentiate within rhombomere 4 (r4), while facial visceral motor neurons differentiate in r5 [2,3]. FBM neurons extend axons via the facial nerve (between embryonic day (E)10.5 and E13.5); concomitantly, neuronal somata migrate caudally from r4 to r6 commencing around E10.5, and finally migrate radially within r6 to form the facial motor nucleus [2,4]. There is very little information about the molecules that drive FBM migration in the mouse embryo. Here we demonstrate for the first time a role for the Wnt/planar cell polarity (PCP) pathway in this process in mammals.

In vertebrates, the PCP pathway has been shown to function during the convergent extension movements of gastrulation and neurulation, and in hair cell polarisation (reviewed by [5,6]). The 'non-canonical Wnts', including Wnt5a, Wnt7a and Wnt11 are implicated in these processes [7-10]. Although the evidence linking Wnts with the PCP pathway is controversial, PCP signalling operates via Frizzleds (Fzs), and the membrane protein VanGogh-like 2 (Vangl2), while the cytoplasmic protein Scribble1 (Scrb1; which has been linked to apico-basal polarity) may also play a role. These components activate Dishevelled, which activates RhoA/Rho kinase (ROCK), and/or Rac and c-Jun amino-terminal kinase (JNK), leading to phosphorylation of molecules involved in cytoskeletal dynamics [11,12]. The Wnt/PCP molecule Vangl2 can bind to Dishevelled, and can signal via both ROCK and JNK [13-16]. In addition, Wnt5a can activate JNK to influence convergent extension [17], and is required for the localisation of Vangl2 in cochlear hair cells [10]. Thus, it is plausible that in FBM migration, JNK and ROCK form part of a signalling cascade containing Wnt5a, Fz, Vangl2, and other PCP components. Other downstream kinases that might function in FBM migration include myosin light chain kinase (MLCK), which is phosphorylated by ROCK and is implicated in cell migration (reviewed by [18]).

While recent (mainly loss-of-function) studies suggest that the Wnt/PCP pathway functions in FBM migration in the zebrafish [19-22], it is unclear which mechanisms operate in mammals (reviewed by [23]). In mice, there are few studies on the mechanisms of FBM migration, although vascular endothelial growth factor (VEGF) has been proposed as a chemoattractant [24]. In this paper, we demonstrate a role of Wnts, other Wnt/PCP components and their downstream signalling pathways in the migration of FBM mammalian neurons.

## Materials and methods

### Animals

For appropriate gestational ages, we counted 0.5 days post-coitus as the formation of a vaginal plug on the following morning after matings. CD1 females were used as the wild-type strain. *Vangl2*, *Scribble*, *Fz3 *and *Wnt5a *homozygous mutants were obtained and genotyped as previously described [10,15,21,25,26]. *Fz3 *mutants were kindly provided by Dr Jeremy Nathans.

### Hindbrain explant cultures

Entire hindbrain explants were cultured on millipore filters (Costar, Fisher, Loughborough, Leicestershire, UK) as previously described [24] and were supplemented with glial cell line-derived neurotrophic factor (R&D systems, Abingdon, Oxfordshire, UK). For chemoattraction affinity bead experiments, Cibacron blue 3AG agarose beads (Sigma-Aldrich, St. Louis, MO, USA) were soaked in phosphate-buffered saline (PBS; control) or in 100 μg/ml of either Wnt5a, Wnt7a, Semaphorin 3A (Sema3A; R&D systems) or VEGF (Peprotech Inc., London, UK) at 4°C overnight, before implantation into hindbrain explants. For the inhibitor experiments, explants were treated with culture medium containing either 10 μM JNK inhibitor (SP 600125, Tocris Bioscience, Bristol UK), 10 μM ROCK inhibitor (Y27632, Calbiochem, UK) or 10 μM MLCK inhibitor (ML-7, Calbiochem). In some cases, inhibitors were applied to hindbrain explants containing Wnt beads to test whether chemoattraction by beads was blocked. For inhibition of Wnt function, a cocktail of secreted frizzed-related proteins (SFRPs; 1, 2 and 3; 500 ng/ml; R&D systems) was applied to explants in the culture medium. Although binding specificities of SFRPs are not completely characterised, SFRP 1 and 2 can bind Wnt5a (for example, see [27]). A combination of SFRPs was applied to block a spectrum of putative Wnt-mediated interactions. After 2 days, explants were fixed with 4% paraformaldehyde for 1 hour and processed for immunohistochemistry.

### Immunohistochemistry and scoring of FBM migration in explants

After fixation, explants were washed several times with PBS containing 1% Triton (PBT) then blocked in PBT containing 10% sheep serum for 30 minutes. Subsequently, the explants were incubated overnight at 4°C with anti-Islet1/2 antibody (4D5; 1:200; Developmental Studies Hybridoma Bank, Iowa City, IA, USA), then washed with PBT for several hours, and afterwards incubated for 1 hour with Cy3 anti-mouse secondary antibody (1:800; Jackson ImmunoResearch Laboratories Inc., West Grove, PA, USA). Finally, the explants were washed with PBT and mounted on slides using Fluor-Save (Calbiochem).

Each explant was scored blind using one of the two following scoring systems. For bead implantation chemoattraction assays, explants were scored as 'attraction' if FBM neurons were deflected from their normal trajectory or as 'no attraction' if the FBM migration stream was unperturbed. Explants containing beads plus inhibitors were scored in the same way, focussing on whether FBM cells were deflected from the migration path, rather than on the migration as a whole. For explants treated with inhibitors, explants were scored on a scale of 1–3, ranging from loss of migration (1) to intermediate loss of migration (2) to normal migration (3). As some neurons had already reached r5 in most explants at the time of culture, we scored loss of migration (1) as a failure of neurons to reach r6 and/or a reduction in the number of neurons that had reached r5. Intermediate loss of migration was scored if explants manifest a phenotype between normal and loss of migration, that is, some neurons having reached r6 but less than in the normal case. In some experiments using inhibitors, the dorsal migration of trigeminal motor neurons in r2/3 was also quantified on a 1–3 scale. For each treatment, the percentage of explants in each category was calculated and compared statistically between control and treated groups (χ-squared test).

### Quantification of FBM migration in explants and in *Wnt5a *mutant embryos

For experiments with control (PBS-treated) beads, Wnt5a-treated beads alone or with JNK or ROCK inhibitors, migration was quantified using the Scion image (NIH image) programme. Confocal images were rendered in black and white and inverted so that black pixels represented migrating neurons. For explants containing beads, a box was drawn containing the beads themselves (located at the r3/4 boundary) and encompassing r4 ipsilateral to the beads up to the midpoint of the floor plate (Additional file 1A). The number of pixels within the box was counted and the mean number of pixels under each condition was presented graphically. For explants without beads and cultured as controls or with inhibitors, a box was drawn over r4 bilaterally and over r6 bilaterally, containing the FBM neuron migratory stream (Additional file 1E). The ratio of r6 pixels/r4 pixels gives an indication of how successfully migration has occurred. The mean ratio was calculated under each condition. For *Wnt5a *mutant embryos and wild-type littermates, the width of the FBM migratory stream was measured at a dorsoventral midpoint using the Scion image programme to quantify the defect. Statistical comparisons were done using a *t*-test.

### *In situ *hybridisation and immunohistochemistry

*In situ *hybridisation on whole-mounts or on cryosections of mouse embryos was performed as previously described [28,29]. Tissues were hybridised with specific digoxigenin-labeled probes for *Islet-1 *(gift from Professor A Simeone, SEMM, Naples, Italy), *Wnt 5a*, *Wnt 7a*, *EphA4 *(gift from Dr U Drescher, MRC Centre of Developmental Neurobiology, London, UK), or *Slit1 *(gift from Dr M Tessier-Lavigne, Genentech, San Francisco, USA).

## Results

### Wnt 5a and Wnt7a attract FBM neurons in a migration assay

**Figure 1:**
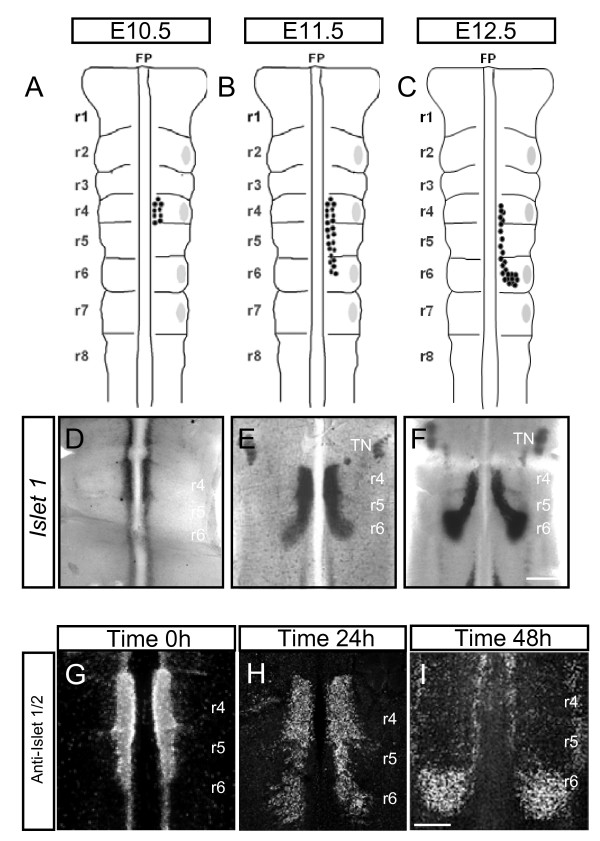
**Spatiotemporal pattern of facial branchiomotor (FBM) neuronal migration *in vivo *and in explants**. **(A-C) **Schematic representations of mouse embryonic hindbrain at embryonic stages (E)10.5 (A), E11.5 (B) and E12.5 (C). FBM neurons are shown by black dots and migrate from rhombomere (r)4 (E10.5) to r6 (E12.5). Grey patches represent dorsal exit points of motor axons. **(D-F)*** Islet-1 in situ *hybridisation on flatmounted hindbrains showing FBM neurons migrating from r4 to r6 at E10.5 (D), E11.5 (E) and E12.5 (F). **(G-I) **Hindbrain explants cultured on filters and immunostained with anti-Islet 1/2 antibody. Time 0 h (G) represents the beginning of culture period when some FBM neurons have started to migrate into r5. Time 24 h (H) shows that more FBM neurons are present in r5 and that some have started to turn dorsally into r6. Time 48 h (I) shows FBM neurons that have reached their final destination in r6 to form a nucleus. TN, trigeminal motor nucleus. Scale bars: 500 μm in (D-F); 250 μm in (G-I).

**Figure 2:**
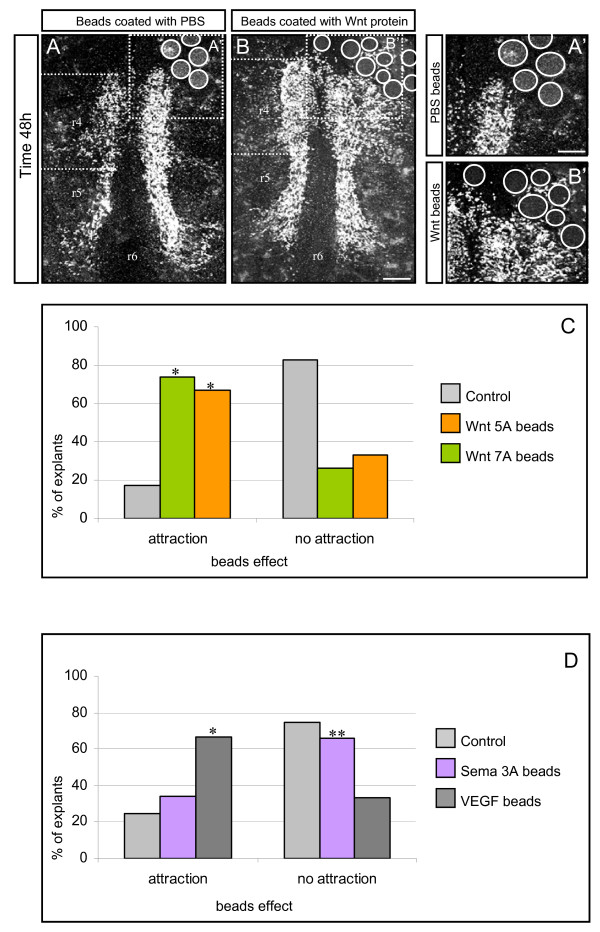
**Chemoattractant effect of Wnt proteins in hindbrain explants**. **(A-B') **Embryonic stage (E)11.5 mouse hindbrain explants with Wnt-coated beads placed in a region of rhombomere (r)4 rostral and lateral to the facial branchiomotor migratory stream: (A) phosphate-buffered saline (PBS) control; (B) Wnt protein; (A', B') higher magnifications of regions in boxes in (A, B), respectively. White circles mark beads. Scale bars: 250 μm in (A, B); 125 μm in (A', B'). **(C, D) **Quantification of bead experiments, with proteins as indicated. (C) Wnt7a versus control *p *< 0.001, Wnt5a versus control *p *< 0.001 (indicated by asterisks), n = 25 explants in each group. (D) Vascular endothelial growth factor (VEGF) versus control *p *< 0.01 (positive control; indicated by single asterisk). Semaphorin 3A (Sema3A) versus control *p *> 0.05 (negative control; indicated by double asterisks), n = 10 explants in each group.

In the mouse embryo, FBM migration occurs between E10.5 and E14.5. FBM neurons are born in r4 from E9.5 onwards; and start migrating at E10.5, reaching r5 by E11.5 and r6 by E12.5 (Figure 1A–C), [2,4]. In r6, neurons migrate laterally,, starting to form a nucleus by E12.5 (Figure 1C), with nucleus formation continuing until E14.5. The migration can be followed by *in situ *hybridisation for *Islet-1*, which is motor neuron-specific at r4-6 axial levels [30] (Figure 1D–F).

In order to investigate the molecular mechanisms of FBM migration, we used a migration assay [24] in which E11.5 mouse hindbrains were isolated and cultured, flattened on filters, for 48 hours. Hindbrains were dissected out early on E11.5, as it was found that isolation of hindbrains on E10.5 led to poor tissue and motor neuron viability. Immunostaining of explants with anti-Islet-1/2 antibody at time 0 showed that most FBM neurons were located in r4, whereas a minority had migrated into r5 (Figure 1G). After 24 hours *in vitro*, many FBM neurons had reached r5 and some had reached r6 and started to turn laterally (Figure 1H). After 48 hours, FBM neurons had reached r6 and coalesced into a characteristic compact nucleus (Figure 1I), reflecting a similar or slightly later developmental stage than that observed using *Islet-1 in situ *hybridisation on E12.5 hindbrains (Figure 1F). Other branchiomotor neurons, such as those of the trigeminal nucleus, which undergo a lateral migration, were also visualised using Islet-1/2 immunostaining (data not shown).

We tested the effects of Wnt proteins on this migration pattern, using beads soaked in Wnt5a or Wnt7a protein (or PBS controls) in E11.5 hindbrain explants cultured for 48 hours. These are 'non-canonical' Wnts, which have been linked to convergent extension movements in fish and frogs (reviewed by [5,31]). When PBS-soaked control beads were placed unilaterally in rostral r4, we found that the FBM migration resembled that in controls, that is, cells were not deflected from their normal migration route (Figure 2A, A'). However, placement of beads soaked in Wnt5a or Wnt7a protein led to a coalescence of FBM neurons around the beads, suggesting that there was a chemoattractant effect (Figure 2B, B'). FBM neurons in r4 and also in r5 migrated laterally, and in some cases cells from the contralateral side also moved across the midline towards the beads. Three-dimensional confocal images of these explants suggested that FBM neurons had collected around the beads. Explants containing either PBS-soaked or Wnt-soaked beads were then scored blind as to whether FBM neurons were deflected from their normal course ('attraction') or not ('no attraction'). Quantification and statistical analysis showed that this effect was significantly different from controls (Figure 2C). We also quantified migration in the presence of Wnt-coated and PBS control beads by pixel counting using the Scion image programme (see Materials and methods; Additional file 1A, B). This method also showed a significant difference between the two groups. In separate experiments, Wnt beads were also presented in r6/r7 to test whether they could attract FBM neurons caudally. An effect was detected, but showed somewhat less clearly that FBM neurons deviated from their pathway than that obtained from rostral placement of beads, possibly because of the proximity to the normal migration route/FBM nucleus (data not shown). However, our data are consistent with a role for Wnts in the caudal and lateral displacement of FBM neurons.

Previous studies have shown that FBM neuron migration is, in part, dependent on VEGF binding to its neuropilin-1 (Npn-1) receptor, whereas the alternative Npn-1 ligand Sema3A plays no role [24]. We therefore used VEGF and Sema3A-coated beads in this assay as positive and a negative controls, respectively. Our results confirmed that while VEGF acted as a chemoattractant, Sema3A had no effect (Figure 2D). These data show that Wnt5a and Wnt7a can chemoattract FBM neurons in hindbrain explants. In our assay, the magnitude of the effect is similar to that of VEGF.

### *Wnt5a *and *Wnt7a *expression patterns in the hindbrain are consistent with a possible role as FBM guidance cues

**Figure 3:**
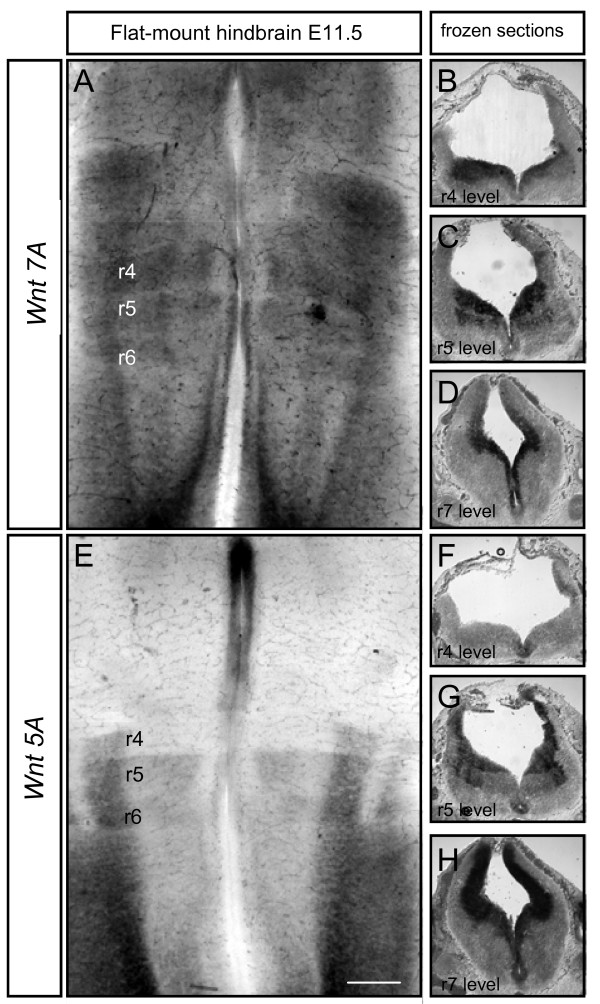
**Expression patterns of *Wnt 7A *and *Wnt 5A *in the hindbrain**. **(A, E) **mRNA *in situ *hybridisation for *Wnt7A *and *Wnt5A *on flat-mounted hindbrains of embryonic stage (E)11.5 mouse embryos, dissected free of all surrounding tissues. **(B-D, F-H) **Cryosections of E11.5 mouse embryo *in situ *hybridised for *Wnt7A *and *Wnt5A *genes, respectively. Rhombomere (r) levels are r4 (B, F), r5 (C, G) and r7 (D, H). Scale bars: 250 μm in (A, E); 500 μm in (B-D, F-H).

In order to determine whether Wnt5a and/or Wnt7a might function as chemoattractants *in vivo*, we analysed their expression profiles in the mouse hindbrain. Whole-mount *in situ *hybridisation at E11.5 showed that in isolated neuroepithelial preparations, *Wnt7a *is expressed throughout the hindbrain, including a dorsal stripe and the floor plate (Figure 3A). Cryosections *in situ*-hybridised for *Wnt7a *mRNA showed that this expression was in the ventricular zone and generally in the neuroepithelium (Figure 3B–D). Intriguingly, whole-mount *in situ *hybridisation for *Wnt5a *showed a different pattern, with a caudal^high ^to rostral^low ^gradient. This was a step gradient rather than a smooth gradient, with changes in expression level coinciding with rhombomere boundaries. There was little or no expression in r2/r3 and r4, apart from the floor plate, higher levels in r5/6 and then increased expression caudally, including a dorsal stripe (Figure 3E). Analysis of *in situ*-hybridised cryosections confirmed that *Wnt5a *forms a gradient in the hindbrain with higher levels caudally (Figure 3F–H). Taken together with the chemoattractant effect of Wnt protein *in vitro*, these expression patterns indicate that Wnt5a in particular could chemoattract FBM neurons along a rostrocaudal migration path *in vivo*, although Wnt7a could play an adjunct role. The rostrocaudal gradient of *Wnt5a *expression demonstrated here is consistent with previous data showing an attractant effect of r5/6 on FBM neurons in heterospecific mouse/chick grafting experiments [32].

### FBM migration is severely disrupted in *Vangl2 *and *Scribble *mutants

**Figure 4:**
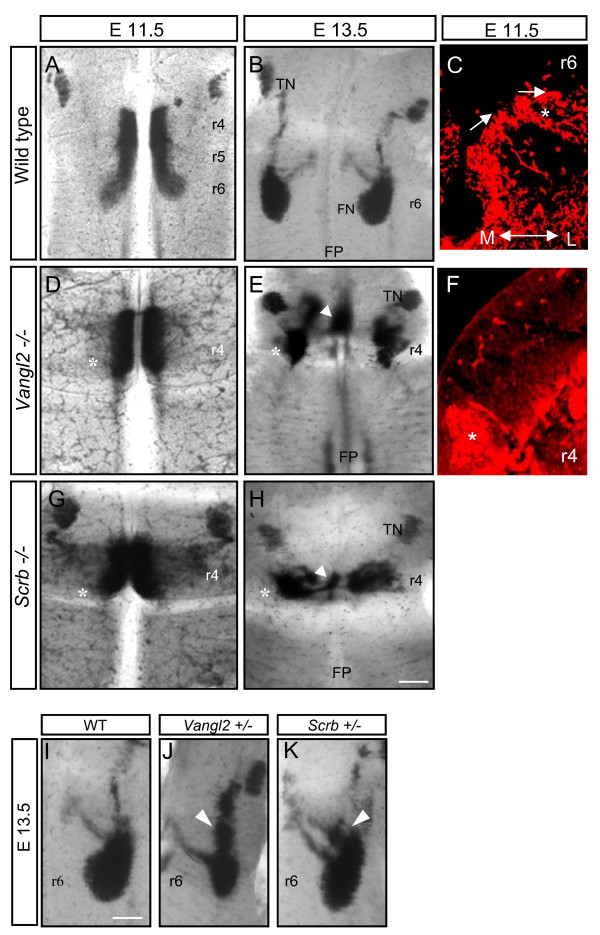
**Analysis of *Vangl2 *and *Scribble *mutant phenotypes**. **(A, B, D, E, G, H, I-K) ***In situ *hybridization with *Islet-1 *probe on flat-mount hindbrains showing facial branchiomotor (FBM) migration in wild-type embryos, and *Vangl2 *and *Scribble *heterozygotes and homozygotes. Embryonic stages (E) and probes are as indicated. Both homozygote mutants present a very dramatic failure of FBM migration (asterisks in (D, E, G, H)) with a population of motor neurons in the floor plate (FP, arrowheads). (J, K) In heterozygous *Vangl2 *and *Scribble *(*Scrb*) littermates at E13.5, a stream of cells arrest in the dorsal region of rhombomere (r)5 (arrowheads) compared to wild type (WT) (C, F) Transverse sections through the hindbrains of wild-type (C) and *Vangl2*-/- (F) hindbrains, immunostained with anti-Islet1/2 antibody to show FBM neurons migrating laterally in r6 (C) to form the FBM nucleus (asterisk) or remaining in r4 (asterisk in F). *(*I). Scale bars: 250 μm in (A, B, D, E, G, H); 100 μm in (C, F); 125 μm in (I-K). TN, trigeminal motor nucleus.

Since it is possible that in this system Wnts operate via, or co-operate with, the PCP signalling pathway, we next analysed loop-tail and circle-tail mice, which are mutant for the *Vangl2 *and *Scribble *genes, respectively [25,33]. These mice manifest neural tube defects, including an open hindbrain. However, observation of flat-mounted preparations showed that normal rhombomere morphology was maintained. mRNA *in situ *hybridisation of mutant hindbrains for *EphA4 *and *Krox-20 *showed that these genes were expressed in r3 and r5, while *Slit1 *was expressed in the floor plate as previously reported [34-36] (Additional file 2A, B, E, F; data not shown). We therefore compared the pattern of FBM neuronal migration using *in situ *hybridisation for *Islet-1 *on wild-type, homozygous and heterozygous *Vangl2 *and *Scribble *hindbrains at E11.5 and E13.5. We observed dramatic defects in FBM migration in the homozygous embryos of both lines compared to wild-type embryos. In wild-type embryo litter-mates at E11.5, there was a normal distribution of FBM neurons, many of which had reached r5 (Figures 1 and 4A). Some had started to migrate laterally in r6 (Figure 4A, C). By contrast, in *Vangl2 *and *Scribble *mutants at E11.5, all FBM neurons had failed to migrate caudally and were still located in r4 (Figure 4D, F, G).

By E13.5 in wild-type embryos (Figure 4B), FBM neurons had clustered in r6 while in both *Vangl2 *and *Scribble *mutants there was a highly abnormal distribution of FBM neurons, with the majority having migrated laterally to form an ectopic facial motor nucleus in dorsal r4 (Figure 4E, H). This migration mode is more characteristic of other populations of hindbrain branchiomotor neurons, for example, trigeminal [37]. Another subpopulation of FBM neurons was located in the centre of the floor plate in r4 (Figure 4E, H). Some heterozygotes of both lines appeared normal, but in a few cases at E13.5 there were FBM neurons in rostral r5 that had diverged from the migratory stream, and the facial motor nucleus appeared less compact compared with wild-type embryos, occupying part of r5 as well as r6 (Figure 4I–K). These observations suggest that the normal caudal migratory path of FBM neurons had been transformed into a lateral or medial migration route in the absence of either of these two PCP genes (Figure 4G–I). The aberrant lateral FBM migration is reminiscent of that seen in *Hoxb1 *mutants at early developmental stages (E11.5), in which mis-specification of the FBM population results in a 'default state' of lateral migration, characteristic of branchiomotor neurons at other axial levels [2,38]. The aberrant medial migration route suggests that some FBM neurons might fail to express receptors for Netrin-1 or Slit, which are normally involved in repelling cranial motor neurons from the floor plate [36,39]. In *Vangl2 *and *Scribble *homozygous embryos, the lateral migration of other branchiomotor neuron populations was normal, for example, trigeminal (Figure 4E, H), suggesting that these genes specifically direct the caudal FBM migration. mRNA *in situ *hybridisation for *Wnt5a *in the *Vangl2 *mutant background showed a normal distribution of transcript, excluding the possibility that the mutant phenotype could be caused by the absence or a change in the expression of *Wnt5a *(Additional file 2C, D).

### Attenuation of Wnt signalling *in vitro *and *in vivo *disrupts FBM migration

**Figure 5:**
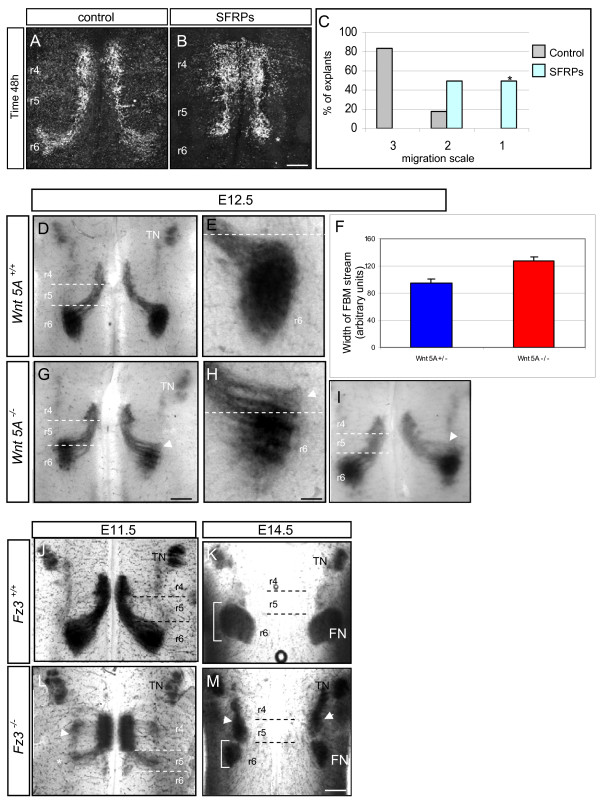
**Analysis of the effects of attenuation of Wnt signalling on facial branchiomotor (FBM) migration**. **(A, B) **Control hindbrain explant (A) and secreted frizzed-related protein (SFRP)-treated explant (B) immunostained for Islet-1/2 after 48 hours *in vitro*. **(C) **Quantification of FBM migration in hindbrain explants treated with SFRPs. SFRP-treated versus control *p *< 0.01 (indicated by asterisk), n = 10 explants in each group. **(D, E, G-M) **Analysis of mutant phenotypes by *in situ *hybridisation with *Islet-1 *probe. (D, E, G-I) Flat-mounted hindbrains of embryonic stage (E)12.5 mouse embryos of *Wnt5A*^+/+ ^(wild type) and homozygote mutant litter-mates *Wnt5A*^-/- ^in which a stream of cells (arrowheads in (G-I)) is located in rhombomere (r)5 and does not reach their final position in r6 compared to wild type (D). (E, H) Higher magnifications of FBM neurons in wild type and homozygote mutant showing a decompacted nucleus in the latter. **(F) **Quantification of the migration defect by measurement of the width of the FBM migratory stream showing this is increased in *Wnt5A *mutants. N = 8; *p *< 0.05 (error bars = sems). (J, L) Flat-mounted hindbrains of E11.5 mouse embryos of *Fz3*^+/+ ^(wild type) and its *Fz3*^-/- ^litter-mate homozygote mutant in which a striking loss of migration of the FBM population can be observed. In the *Fz3*^-/- ^mutant a much reduced stream of cells migrated caudally into r5 (asterisk) and there is aberrant dorsolateral migration of FBM neurons in r4 (arrowhead). (K, M) At E14.5, FBM neurons in homozygote *Fz3*^-/- ^hindbrains (M) contain an FBM nucleus in r6 that is much smaller than in the wild-type (K) and there are ectopic dorsal nuclei in r4 and r5 (arrowheads). Brackets in (K, M) indicate the smaller size of the FBM nucleus in *Fz3*-/- mutant. Scale bars: in (B), 250 μm and applies to (A, B); in (G), 375 μm and applies to (D, G, I,); in (H), 125 μm and applies to (E, H); in (M), 375 μm and applies to (J, M). FN, facial nucleus; TN, trigeminal nucleus.

SFRPs are known to interfere specifically with Wnt-Fz-dependent processes (reviewed by [40]) and can be used to establish an intrinsic function of Wnts. We applied a combination of SFRPs 1–3 to hindbrain explants at the beginning of the culture period. The majority of explants showed severe disruption of the caudal FBM migration, compared with control explants (Figure 5A, B). Explants were scored on a scale of 1–3, ranging from complete loss of migration (1) to intermediate loss of migration (2) to normal migration (3). A comparison of values in untreated versus SFRP-treated explants showed a significant shift in values towards a loss of migration, suggesting that SFRPs strongly inhibit this process (Figure 5C). A caveat to this interpretation may be that SFRPs can, under some circumstances, bind to Fzs and act as guidance cues [41]. However, we believe that, taken together with data from *Wnt *and *Fz *mutant embryos (see below), our results suggest that endogenous Wnt-Fz interactions drive FBM migration.

We also analysed *Wnt7a *and *Wnt5a *mutant mouse embryos, by performing mRNA *in situ *hybridisation for *Islet-1 *on E11.5 hindbrains. In *Wnt7a *mutant embryos, the distribution of FBM neurons closely resembled that in wild-type litter-mates, suggesting that there was no defect (data not shown). However, *Wnt5a *mutants showed a partial defect in the migration, with some divergent streams of migrating cells, resulting in a broader and less compact FBM nucleus extending into r5 as well as r6, suggesting that some of the FBM neurons had stalled (Figure 5D, E, G–I). This phenotype resembled the phenotype of heterozygous *Vangl2 *and *Scribble *mutant embryos (Figure 4J, K). *Wnt5a *heterozygous embryos showed no defects and were indistinguishable from wild-type embryos (Figure 5D; data not shown).

In order to quantify the *Wnt5a *mutant defect, we measured the width of the FBM migratory stream at a mid-point between the ventral edge of the migratory stream and the dorsal edge of the nucleus. This method revealed a significant increase in the width of the migratory stream in *Wnt5a *mutants compared with heterozygous embryos (Figure 5F), consistent with the decompaction of the nucleus observed. The phenotype that we observe in *Wnt5a *mutants suggests that *Wnt5a *plays a role in FBM migration *in vivo*, although we hypothesise that other Wnts might also play a role.

A candidate receptor to mediate the effect of Wnt in our system is Fz3, which is expressed in the mouse hindbrain at relevant developmental stages [40]. Fz3 is involved in hair cell polarity in the mouse and its homologue, Fz3a, is implicated in FBM migration in the zebrafish [21,42,43]. We investigated the migration pattern of FBM neurons in *Fz3 *mutants [44] by *Islet-1 in situ *hybridisation as above. We found that there were striking defects in FBM migration in E11.5 and E14.5 homozygous *Fz3 *mutants compared with their wild-type or heterozygous littermates. At E11.5 the majority of FBM neurons had failed to migrate caudally out of r4 and remained close to the midline (Figure 5L, compare with Figure 5J). A reduced number of FBM neurons could be observed migrating caudally, but many neurons also formed streams migrating laterally in r4 and r5. At E14.5 the FBM nucleus was well-formed in wild-type embryos and was located in r6 (Figure 5K). In *Fz3 *mutants, an FBM nucleus was present in r6, but it was strongly reduced in size (Figure 5M). The presence of ectopic nuclei in dorsal r4 and r5 suggests that these originated from the dorsally migrating FBM neurons observed at E11.5. Thus, in *Fz3 *mutants, FBM migration shows severe defects consistent with the involvement of Fz signalling in this system. Attenuation of this pathway leads to many FBM neurons following a lateral 'default' migration pathway, in the manner of other hindbrain branchiomotor neuron subpopulations.

### JNK and ROCK kinases signal during FBM migration and are required for the attractant effects of Wnts

**Figure 6:**
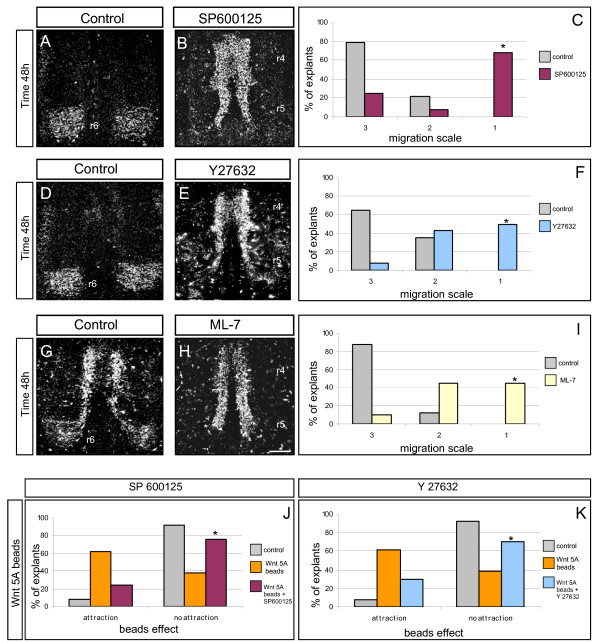
**Inhibition of facial branchiomotor (FBM) migration and of Wnt-mediated attraction**. **(A, D, G) **Control explants cultured for 48 hours and immunostained with anti-Islet 1/2 antibody with FBM neurons located in their final position forming the facial motor nuclei in rhombomere (r)6. **(B, E, H) **Explants treated with c-Jun amino-terminal kinase (JNK) inhibitor (SP600125), Rho kinase (ROCK) inhibitor (Y27632) and myosin light chain kinase inhibitor (ML-7) as labelled. Scale bars: 250 μm (A, B, D, E, G, H). **(C, F, I) **Quantification of the effects of inhibitors on FBM migration in explants. The x-axis is the migration scale where 1 = loss of migration, 2 = intermediate loss of migration, 3 = normal migration. The y-axis is the percentage of explants in a particular category. Inhibitor-treated versus control *p *< 0.001 (indicated by asterisk). **(J, K) **Quantification of the effect of inhibitors on Wnt5A attractive beads. The attractive effect of Wnt5A ectopic beads is inhibited by adding inhibitors SP600125 (J) and Y27632 (K). Inhibitor-treated versus control *p *> 0.05 (indicated by asterisk). (C, F, I, J, K) N = 15–20 explants in each group.

In order to dissect the signalling pathways involved in FBM migration, we applied pharmacological inhibitors of candidate molecules to the FBM migration assay. Inhibitors were applied at the beginning of the culture period and the distribution of Islet-1/2-positive FBM neurons was compared between inhibitor-treated explants and those treated with vehicle alone (DMSO controls). Migration was scored on a scale of 1–3 as for SFRP-treated explants. Explants treated with JNK inhibitor (SP600125) showed a dose-dependent disruption of migration, with 5 μM resulting in a moderate inhibition (57.2% of explants were grade 1 (loss of migration); data not shown) and 10 μM producing an effect in which FBM neurons stopped in r5 (68% of explants were scored as grade 1; Figure 6A–C). Inhibitors of ROCK (Y27632) or its downstream target MLCK (ML-7) both caused FBM migration defects, but in fewer cases, with 40–50% of explants showing grade 1 morphology (Figure 6D–I).

To discover whether these inhibitors produced a general block of neuronal migration in the hindbrain, or had specific effects on the FBM caudal migration, we also assessed their effects on laterally migrating trigeminal (branchiomotor) neurons. JNK and ROCK inhibitors had modest effects in inhibiting lateral migration, though these effects were considerably less than those on FBM neurons' caudal migration (Additional file 3A, B). However, inhibition of MLCK produced effects that were more pronounced (45% of explants at grade 1) and equivalent to those on the FBM caudal migration (Additional file 3C). These data suggest that while MLCK plays a general role in cranial motor neuron migration, JNK and ROCK show specificity in functioning within the FBM caudal migration. As MLCK is a direct target of ROCK, this suggests that MLCK function in the dorsal migration might also be regulated by other upstream components. We also tested whether JNK and ROCK inhibitors blocked FBM neuron attraction by VEGF. Scoring of explants indicated that these inhibitors failed to block attraction by VEGF beads, indicating that JNK and ROCK are specific to Wnt-mediated FBM migration (Additional file 1C, D).

In view of the fact that Wnt5a is the most promising candidate to be an FBM chemoattractant *in vivo*, we next tested whether JNK or ROCK functioned downstream of Wnt5a, by applying inhibitors to explants containing implanted Wnt5a beads. The attractant effect of Wnt5a beads was specifically blocked by adding 10 μM of JNK inhibitor or of ROCK inhibitor (Figure 6J, K). This effect was further quantified by pixel counting (Materials and methods) of the number of FBM neurons in r4 adjacent to Wnt5a beads alone or in the presence of inhibitors. This showed a significant reduction in the number of FBM neurons adjacent to Wnt beads when explants were treated with inhibitors compared with those containing Wnt5a beads alone (Additional file 1B). This suggests that the effect of Wnt5a in FBM migration is mediated by the JNK and ROCK kinases.

## Discussion

We here propose that Wnt5a/7a act as guidance cues/chemoattractants in mammalian neuronal migration. Wnts have previously been implicated in neuronal migrations in *Caenorhabditis elegans*, where they act as chemorepellents [45,46]. Although PCP components play a role in FBM migration in zebrafish [19-22], no Wnt ligands have so far been linked with the migration (reviewed by [23]). In the mouse, correlative data have showed that in *Tbx20 *mutant mice with aberrant FBM neuron migration, several Wnt/PCP components are down-regulated [47]. Our study provides functional data suggesting a role for Wnt signalling via a non-canonical pathway, probably involving PCP components, in mammalian FBM migration.

### Wnt5a/7a act as guidance cues/chemoattractants

In vertebrates, a role for Wnt5a in cell migration is indicated by the finding that down-regulation of Wnt5a impedes cell migration in a wound-healing assay [48]. Our findings add to a growing body of evidence suggesting that the 'non-canonical Wnts', including Wnt5a, Wnt7a and Wnt11, function in aspects of PCP, including convergent extension movements (for example, [7,8]), and cochlear hair cell orientation [9,10]. Intriguingly, *Wnt11 *is also expressed in FBM neurons, and is absent in *Tbx20 *mutants [47]. It is possible, therefore, that several Wnts collaborate during the migration. We found that *Wnt7a *mutant mice had no deficit in FBM migration, whereas in *Wnt5a *mutants, the migration pattern and nuclear formation were impaired. The relatively weak phenotype in *Wnt5a *mutants, however, suggests that whereas Wnt5a plays an important role, Wnt7a and/or other Wnts might play an adjunct role. This idea has a parallel in studies demonstrating that Wnt7a and Wnt5a both pattern cochlear hair cell polarisation, but only *Wnt5a *mutants show cochlear defects [9,10]. In our system the more uniform expression pattern of *Wnt7a *might not rule out a possible role in the migration, as in zebrafish, several other Wnt/PCP components that play a functional role in FBM migration are widely expressed in the hindbrain. Interestingly, in the zebrafish, no Wnt ligands have so far been linked to the FBM migration (reviewed by [23]). It remains a formal possibility in the mouse hindbrain that, as has been suggested in other systems, activation of the PCP pathway might depend on a gradient of Fz activity without involving a Wnt ligand [5,6].

Previous studies on axon guidance in vertebrates have pointed to a role for Wnts in chemoattraction and chemorepulsion of commissural axons and corticospinal axons, respectively, along the rostrocaudal axis of the spinal cord [26,49]. In both cases Wnts (4 and 1/5a) were proposed to be distributed in a rostral^high ^to caudal^low ^gradient, the opposite polarity to that of *Wnt5a *expression in the hindbrain. It is likely, therefore, that local differences in Wnt gradients along the rostrocaudal axis, coupled with utilisation of different receptors/receptor complexes, dictate different cellular responses. In our proposed model, it is possible that endogenous SFRPs also play a role; SFRP2 is expressed in r4 in the mouse E10.5 hindbrain [50], and might enhance the caudal to rostral Wnt gradient by sequestering Wnt rostrally.

Another reason for the relatively modest phenotype in *Wnt5a *mutants is likely to be the role played by VEGF in FBM migration. VEGF is expressed in the floor plate and in a lateral stripe in the mouse hindbrain at E12.5, as well as in a group of cells lateral to the FBM nucleus [24]. VEGF acts as a chemoattractant when presented on beads in an explant FBM migration assay [24] and the present study). VEGF and Wnt signalling are therefore likely to co-operate during the process of FBM migration. As Wnt5a is expressed in a rostro-caudal gradient and VEGF is expressed laterally, it may be that Wnts and VEGF play a role in the caudal and the lateral cell displacements, respectively. It is as yet unclear whether they utilise common signalling pathways; our experiments suggest that VEGF does not act via the JNK and ROCK kinases

### The PCP pathway is implicated downstream of Wnts

**Figure 7:**
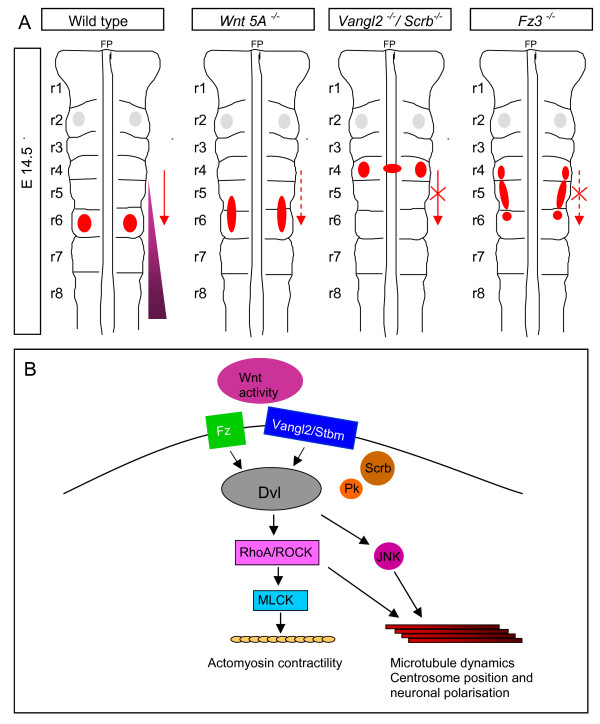
**Schematic diagram of facial branchiomotor (FBM) migration and signalling pathways**. **(A) **Schematic diagrams of the final position of the FBM nucleus in embryonic stage (E)14.5 mouse embryos of wild-type, and *Wnt5a*, *Vangl2*/*Scribble *and *Fz3 *mutant mice. Red patches represent facial nuclei at their final location in wild-type or mutant background. Grey patches show trigeminal nuclei. The red arrow shows the direction of FBM migration in wild-type; the dashed red arrow represents a partial loss of migration in the *Wnt 5A*^-/- ^mutant; crossed red arrow represents a total loss of migration in the *Vangl2/Scrb*^-/- ^mutant; the crossed red dashed arrow indicates a loss of migration and partial lateral FBM migration in r5 and r6 in the *Fz3*^-/- ^mutant. The violet gradient represents the putative distribution of Wnt 5A protein. FP, floor plate. (B) Schematic diagrams of putative Wnt signalling pathways to the cytoskeleton in FBM neurons. Dvl, Dishevelled; Fz, Frizzled; JNK, c-Jun amino-terminal kinase; MLCK, myosin light chain kinase; Pk, Prickle; ROCK, Rho kinase; Scrb, Scribble; Stbm, Strabismus; Vangl2, Van Gogh-like 2.

The striking phenotypes of *Vangl2 *and *Scribble *mutants, in which FBM migration is arrested, add to a growing body of evidence implicating Wnt/PCP components in FBM migration (Figure 7A, B). The defects we describe are closely similar to those previously shown in the zebrafish *Vangl2*/*Trilobite *and *Scribble *mutants [19,20,51]. In zebrafish, in addition to *Vangl2/Trilobite *and *Scribble*, mutation of the Wnt/PCP components *Prickle*, *Fz3a *and *Celsr2 *result in FBM migration defects [19,21,52]. This raises the likely possibility that mice defective for PCP genes such as *Celsr1 *[53], which show convergent extension and hair cell polarity defects, might also show aberrant FBM migration.

It appears that at least one Fz protein, Fz3, functions in FBM migration. In *Fz3 *mutants, as in *Vangl2 *and *Scribble *mutants, the majority of FBM neurons migrated laterally (Figure 7A). *Fz3 *is globally expressed in the mouse hindbrain [54], and in zebrafish, *Fz3a *has been shown to act non-cell autonomously to control FBM migration [21]. Although we cannot distinguish between a cell autonomous or non-cell autonomous mechanism of action of Fz3 in our system, the two mechanisms may not be mutually exclusive. Fz3 might function in chemoattraction of FBM neurons in a cell-autonomous manner, but a Fz activity gradient in the neuroepithelium might also play a role. This would not exclude the possibility that other Fzs might be involved. Fz4 and Fz7 can interact with Wnt5a, and Fz7 is expressed by FBM neurons [47,55]. Other studies link the alternative Wnt receptor Ror2 to Wnt5a-induced cell migration [56]. The possibility of an association between Fz and Vangl2 is also suggested by the observation that a multi-PDZ containing protein, MAGI-3, can bind Fz4 and Fz7 and Vangl2, providing the basis of a receptor complex for JNK signalling [57].

### JNK and ROCK kinases play a role in FBM migration

Our investigation of downstream signalling in FBM migration by the use of inhibitors revealed a most striking role for JNK, both in the migration *per se*, and in the deflection of FBM neurons by the presentation of ectopic Wnt5a protein on beads. ROCK inhibition had a less striking effect on the migration pattern, but could also block the Wnt5a effect. Inhibition of MLCK disrupted FBM migration equivalently to ROCK inhibition, but also inhibited the dorsal migration of other hindbrain branchiomotor neurons, suggesting that it does not function specifically in the rostrocaudal migration route. Both JNK and ROCK are well-known to function downstream in the PCP pathway (reviewed by [12,31]; see Figure 7B for schema). Wnt5a and the PCP pathway have also been linked to both JNK and ROCK in convergent extension movements in fish and frogs [17,58-60]. However, in mammals, PCP downstream signalling pathways have been less clear. Whereas Wnt5a activates JNK in cultured mammalian cells [17,55], a study focussing on convergent extension in the mouse found a role for RhoA-ROCK, but not JNK, downstream of Vangl2 [15].

We cannot completely rule out the possibility that JNK and ROCK function independently of the PCP pathway in our system. However, on balance, our findings suggest that both JNK and ROCK are important effectors of Wnt5a signalling in FBM neurons. By contrast, MLCK may play a role as a common target for several signalling pathways involved in the migration of branchiomotor (including FBM) neurons. A major target of MLCK is myosin II, which localises to the rear of the nucleus during neuronal migration [61,62]; mice mutant for *myosin IIB *show defective facial motor nucleus formation [63,64], consistent with our MLCK inhibitor studies. The downstream targets of JNK and ROCK phosphorylation in FBM neurons are unknown. Promising candidates are microtubule-associated proteins (MAPs); for example, known targets of JNK are the MAPs MAP1B and doublecortin [65,66].

Finally, it will be intriguing for future studies to discover whether Wnt5a or other Wnts orchestrate neuronal migrations or axon projection patterns along the rostrocaudal axis of the hindbrain.

## Conclusion

In this paper we demonstrate that the Wnt/PCP pathway functions in mammalian FBM migration via an attractant mechanism. Both *Wnts *are expressed in the hindbrain, and *Wnt5a *is expressed in a rostral^low ^to caudal^high ^gradient consistent with a chemoattractant role. An *in vivo *role for Wnts is suggested by the finding that attenuation of endogenous Wnt signalling using SFRPs, or by genetic loss of function of *Wnt5a*, disrupts the FBM migration. Genetic deletion of the Wnt/PCP genes *Fz3*, *Vangl2 *and *Scribble *leads to severe disruption of FBM migration. We further show that the JNK and ROCK kinases are downstream components of Wnt signalling in FBM neurons. Our study is the first implicating Wnt/PCP signalling in mammalian FBM neuron migration.

## Abbreviations

E: embryonic day; FBM: facial branchiomotor; Fz: Frizzled; JNK: c-Jun amino-terminal kinase; MAP: microtubule-associated protein; MLCK: myosin light chain kinase; PBS: phosphate-buffered saline; PBT: phosphate-buffered saline containing Triton-X100; PCP: planar cell polarity; r: rhombomere; ROCK: Rho kinase; Scrb: Scribble; Sema3A: Semaphorin 3A; SFRP: secreted frizzed-related protein; Vangl2: Van Gogh-like 2; VEGF: vascular endothelial growth factor.

## Competing interests

The authors declare that they have no competing interests.

## Authors' contributions

VV performed all the experiments reported in this paper and developed the concepts. SG is the senior author, in whose laboratory the work was done, and who conceived the project together with MS, a co-grant holder for the project. PC, DQ, AD and MK provided *Wnt5a *mutant mice, while NS provided *Frizzled3 *mutant mice.

## Supplementary Material

Additional file 1 **Quantification of FBM migration in explants containing Wnt- or PBS-treated beads. (A) **Example of FBM neurons labelled using anti-Islet-1/2 antibody in control explant cultured for 48 hours. Position of beads shown by white circles. Area quantified by pixel counting is shown by the red box and encompasses the beads themselves located at the r3/r4 boundary, and the ipsilateral r4 up to the midline. **(B) **Quantification of pixels adjacent to Wnt5a or control beads, or Wnt5a beads in the presence of JNK inhibitor SP600125 or ROCK inhibitor Y27632. N = 5 explants in each case. Statistical comparison of control versus Wnt5a *p *< 0.01 (indicated by asterisk); Wnt5a versus SP600125 *p *< 0.01 (indicated by asterisk); and Wnt5a versus Y27632, *p *< 0.05 (indicated by asterisk). **(C) **Scoring of explants containing VEGF-treated beads and VEGF-treated beads with Y27632 inhibitor. N = 6–10 explants; *p *> 0.05. **(D) **Scoring of explants containing VEGF-treated beads and VEGF-treated beads with SP600125. N = 6–10 explants; *p *> 0.05. **(E) **Example of control explant cultured for 48 hours showing boxes (outlined in red) in r4 and r6 used for pixel counting to quantify migration. Ratio of r6 pixel counts/r4 pixel counts was derived for each explant. **(F) **Quantification of the mean ratio of pixels r6/r4 for control explants and explants treated with SP600125 and Y27632 inhibitors. Control versus SP600125 *p *< 0.01 (indicated by asterisk); control versus Y27632 *p *< 0.05 (indicated by asterisk). Scale bars: 250 μm in (A); 300 μm in (E). Click here for file

Additional file 2 **Expression pattern of genes in *Vangl2 *and *Scribble *mutants**. **(A, B) **Flat-mounted hindbrains of E11.5 mouse embryos *in situ*-hybridised for *EphA4 *in wild type (A) and its *Scrb*^-/- ^homozygous mutant litter-mate (B), showing that rhombomere segmentation is conserved in the mutant background. **(C-F) **Transverse cryosections of E11.5 wild type and *Vangl2*^-/- ^homozygous mutants at the r4 level, *in situ *hybridised for *Wnt5A *(C, D) and *Slit1 *probes (E, F), showing that no difference in expression pattern was observed. Scale bars: 250 μm (A, B); 500 μm (C-F). Click here for file

Additional file 3 **Quantification of the dorsal migration of trigeminal branchiomotor neurons in explants treated with inhibitors. **The dorsal migration of trigeminal motor neurons was scored on a 1–3 scale as for the FBM migration. Axes as in Figure 6. **(A) **JNK inhibitor SP600125, **(B) **ROCK inhibitor Y27632, **(C) **MLCK inhibitor ML-7. Inhibitor-treated versus control *p *> 0.05 (A, B), *p *< 0.001 (indicated by asterisk) (C). N = 20 explants in each group. Click here for file
