# Supplementary figures and images for: Wnt activity guides facial branchiomotor neuron migration, and involves the PCP pathway and JNK and ROCK kinases
Source: Neural Dev. 2009 Feb 11;4:7. doi: 10.1186/1749-8104-4-7 (PMC2654884; doi:10.1186/1749-8104-4-7)

Suppl. Fig. 1

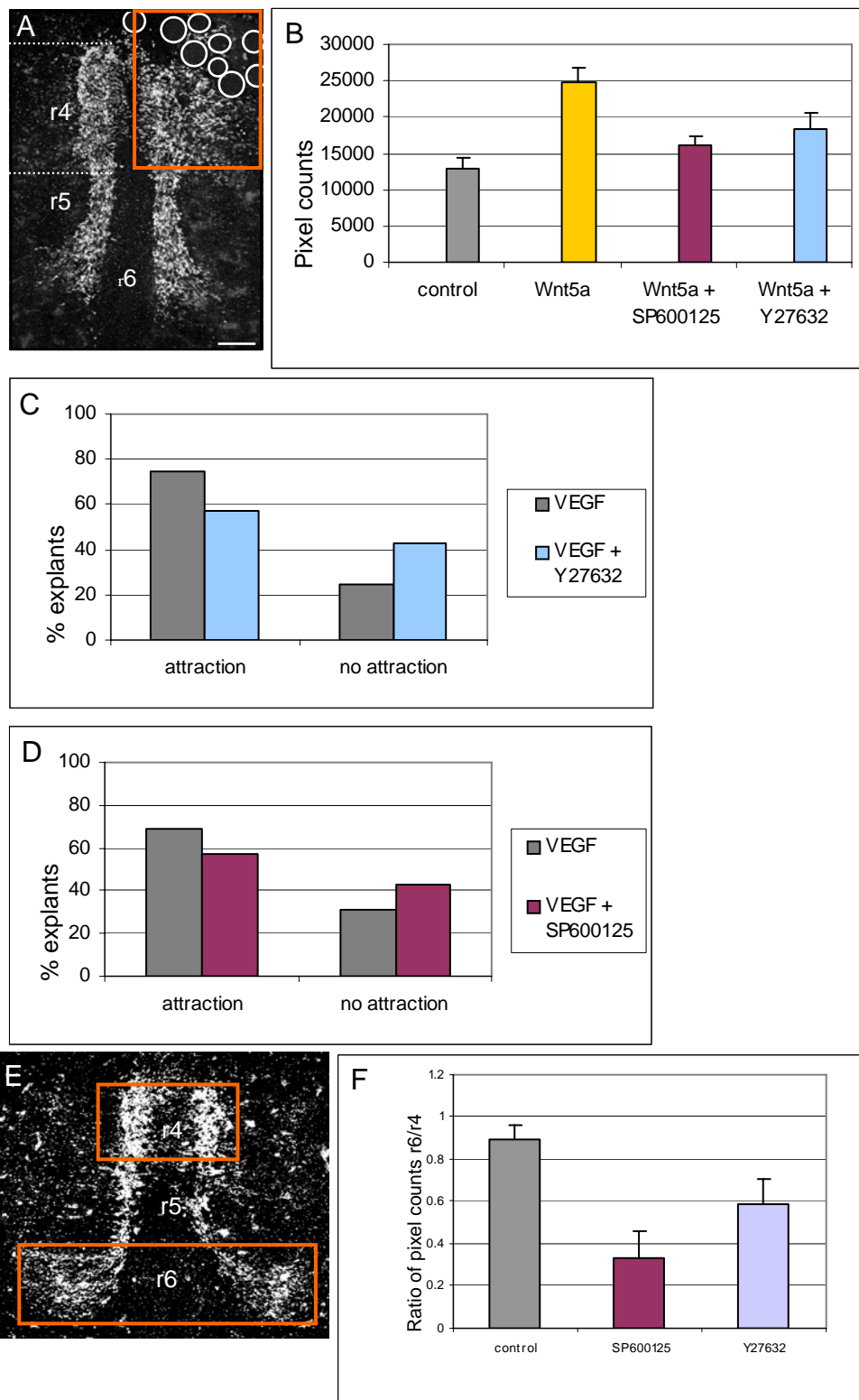

Supplement: Additional file 1 — Quantification of FBM migration in explants containing Wnt- or PBS-treated beads. (A) Example of FBM neurons labelled using anti-Islet-1/2 antibody in control explant cultured for 48 hours. Position of beads shown by white circles. Area quantified by pixel counting is shown by the red box and encompasses the beads themselves located at the r3/r4 boundary, and the ipsilateral r4 up to the midline. (B) Quantification of pixels adjacent to Wnt5a or control beads, or Wnt5a beads in the presence of JNK inhibitor SP600125 or ROCK inhibitor Y27632. N = 5 explants in each case. Statistical comparison of control versus Wnt5a p < 0.01 (indicated by asterisk); Wnt5a versus SP600125 p < 0.01 (indicated by asterisk); and Wnt5a versus Y27632, p < 0.05 (indicated by asterisk). (C) Scoring of explants containing VEGF-treated beads and VEGF-treated beads with Y27632 inhibitor. N = 6–10 explants; p > 0.05. (D) Scoring of explants containing VEGF-treated beads and VEGF-treated beads with SP600125. N = 6–10 explants; p > 0.05. (E) Example of control explant cultured for 48 hours showing boxes (outlined in red) in r4 and r6 used for pixel counting to quantify migration. Ratio of r6 pixel counts/r4 pixel counts was derived for each explant. (F) Quantification of the mean ratio of pixels r6/r4 for control explants and explants treated with SP600125 and Y27632 inhibitors. Control versus SP600125 p < 0.01 (indicated by asterisk); control versus Y27632 p < 0.05 (indicated by asterisk). Scale bars: 250 μm in (A); 300 μm in (E). [file 1749-8104-4-7-S1.pdf]

Suppl. Figure 2

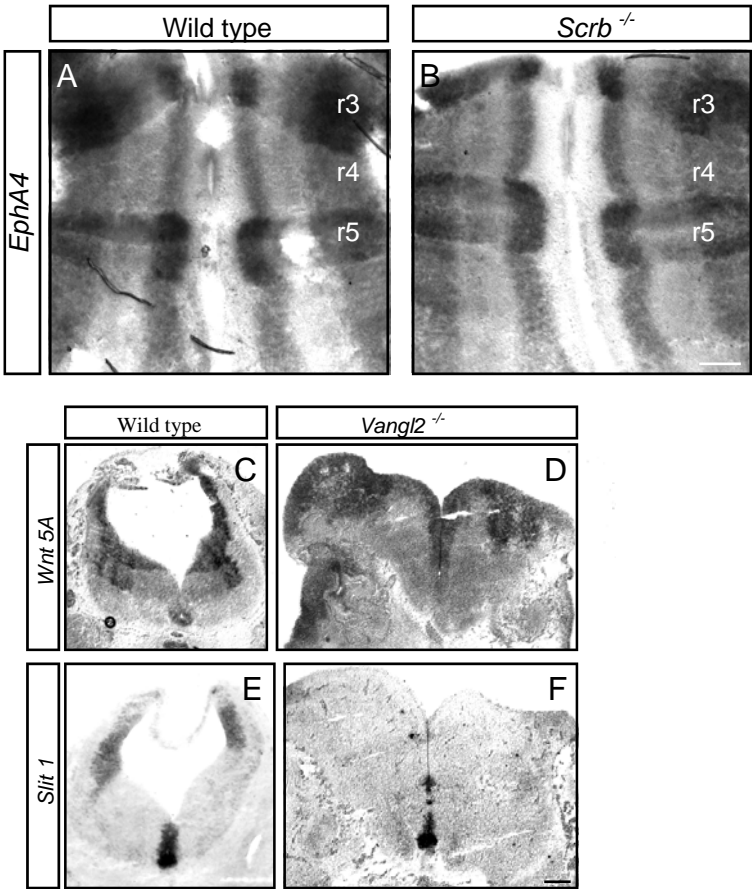

Supplement: Additional file 2 — Expression pattern of genes in Vangl2 and Scribble mutants. (A, B) Flat-mounted hindbrains of E11.5 mouse embryos in situ-hybridised for EphA4 in wild type (A) and its Scrb-/- homozygous mutant litter-mate (B), showing that rhombomere segmentation is conserved in the mutant background. (C-F) Transverse cryosections of E11.5 wild type and Vangl2-/- homozygous mutants at the r4 level, in situ hybridised for Wnt5A (C, D) and Slit1 probes (E, F), showing that no difference in expression pattern was observed. Scale bars: 250 μm (A, B); 500 μm (C-F). [file 1749-8104-4-7-S2.pdf]

Suppl. Figure 3

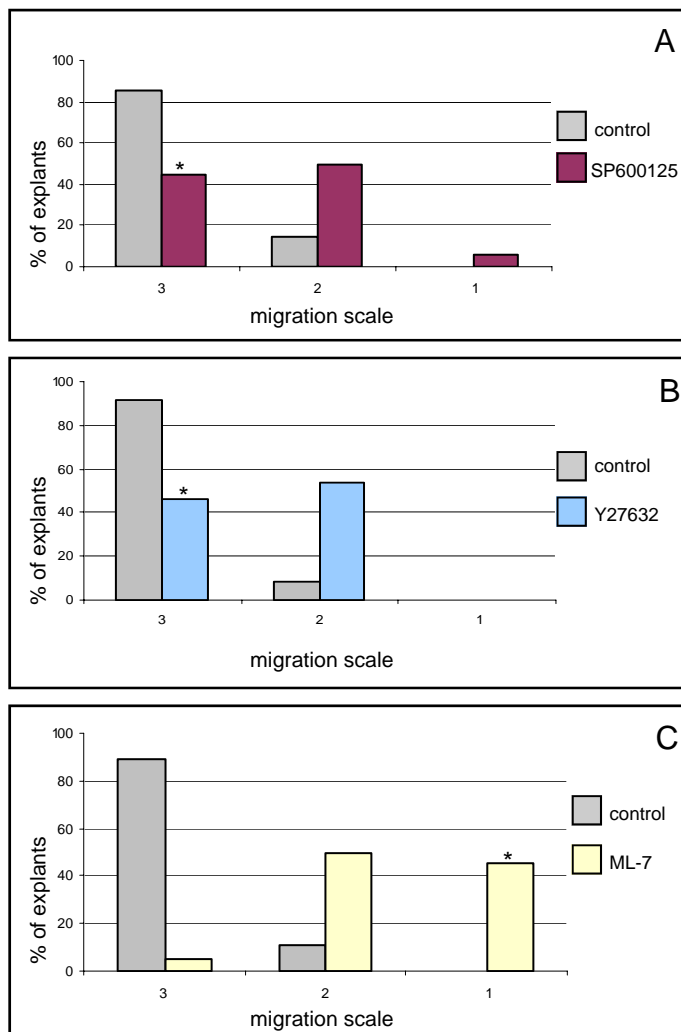

Supplement: Additional file 3 — Quantification of the dorsal migration of trigeminal branchiomotor neurons in explants treated with inhibitors. The dorsal migration of trigeminal motor neurons was scored on a 1–3 scale as for the FBM migration. Axes as in Figure 6. (A) JNK inhibitor SP600125, (B) ROCK inhibitor Y27632, (C) MLCK inhibitor ML-7. Inhibitor-treated versus control p > 0.05 (A, B), p < 0.001 (indicated by asterisk) (C). N = 20 explants in each group. [file 1749-8104-4-7-S3.pdf]
